# Establishment of Prediction Equations of Lean Body Mass Suitable for Chinese Adults

**DOI:** 10.1155/2019/1757954

**Published:** 2019-06-24

**Authors:** Jinhua Li, Jingjie Shang, Bin Guo, Jian Gong, Hao Xu

**Affiliations:** ^1^Department of Ultrasound, Medical Imaging Center, The First Affiliated Hospital, Jinan University, Guangzhou, China; ^2^Department of Nuclear Medicine, The First Affiliated Hospital, Jinan University, Guangzhou, China

## Abstract

**Aim:**

To develop predictive equations of lean body mass (LBM) suitable for healthy southern Chinese adults with a large sample. LBM measured by dual-energy X-ray absorptiometry (DXA) are considered as the standard ones.

**Methods:**

Retrospective analysis was conducted on the consecutive people who did total body measurement with DXA from July 2005 to October 2015. People with diseases that might affect LBM were excluded and overall 12,194 subjects were included in this study. Information about the 10,683 subjects (2,987 males and 7,696 females) from July 2005 to November 2014 was used to establish equations. These subjects were grouped by sex and then subdivided according to their body mass index (BMI). The female group was divided into another two subgroups: the premenopausal and postmenopausal subgroups. Equations were developed through stepwise multilinear regression analysis of height, weight, age, and BMI. Information about the 1,511 subjects (395 males and 1116 females) from December 2014 to October 2015 was used to verify the established equations.

**Results:**

BMI, height, weight, and age were introduced into the equations as independent variables in the male group, while age was proved to have no influence on LBM in the female group. Regrouping according to BMI or menopause did not increase the predictive ability of equations. Good agreement between LBM evaluated by equation (LBM_PE) and LBM measured by DXA (LBM_DXA) was observed in both the male and female groups.

**Conclusion:**

Predictive equations of LBM suitable for healthy southern Chinese adults are established with a large sample. BMI was related to LBM content; however, there is no need for further group based on BMI or menopause while developing LBM questions.

## 1. Introduction

Lean body mass (LBM) accounts for most of the human body and is known to be one of the main drivers of energy expenditure. It plays an important role in many physiological and pathological processes and is a major predictor of body functions, morbidity, and mortality [1]. Reduction in LBM may have negative effects on many health outcomes. Evaluation of LBM is an important way of assessing nutritional and health status and predicting diseases in both clinical and research settings, helping get more accurate evaluation of the efficacy, side effects, and dosage of medicines [2–4]. Moreover, LBM is even more important for the elderly population because ageing is related to substantial decrease of LBM [5].

Common LBM evaluation techniques in clinical settings include bioelectrical impedance analysis (BIA), magnetic resonance imaging (MRI), and dual-energy X-ray absorptiometry (DXA). BIA has simple operation and high speed, but its accuracy is relatively lower than that of the other two. Though MRI and DXA are highly accurate, they are hardly used in large-scale epidemiologic studies and in remote areas because of complicated operation and high cost [6]. A regression equation designed based on morphological statistics, such as height and weight, is simple, fast, and inexpensive in calculating LBM and especially suitable for studies with large samples. Estimating equation is a way of calculating LBM when DXA or MRI is not available [7].

Differences in LBM between different races have been observed [8, 9]. Not only is the body composition of different continents' inhabitants different [10], but also even people from different Asian ethnic groups have different body composition [9, 11]. As researchers increase their understanding of the influencing factors of LBM, it is urgent to develop race-specific estimating equations for LBM. A number of researchers previously developed anthropometric prediction equations for estimation of LBM. However, most of them estimated LBM indirectly by estimating the percentage of body fat content [10, 12, 13], while only a few studies predicted LBM with specifically developed equations [5, 7, 14, 15]. Moreover, the LBM equations were for white, European, and Asian populations, and no one only for the Chinese people has been developed. Wen et al. [16] developed an anthropometric prediction equation for Chinese adults' limb muscle mass in 2011. However, there is no anthropometric prediction equation to evaluate LBM of the whole body for Chinese citizens. Therefore, we have to use LBM equation designed based on the characteristics of other countries' populations. For example, the most commonly used LBM equation in evaluating treatment effects on patients with tumors using positron emission tomography is tailored to Caucasians [17].

This study aimed to develop simple anthropometric equations which would make estimation of LBM in both clinical and epidemiological settings and monitoring of southern Chinese people's LBM easier. Furthermore, we validated our equations and analyzed the effects of body mass index (BMI) and menopause on equation development.

## 2. Materials and Methods

This study was approved by the Ethics Committee of the First Affiliated Hospital of Jinan University ([2019] Ethics Approval Section No. 017) and conducted in accordance with the basic principles of the Declaration of Helsinki. All the participants provided written informed consent.

### 2.1. Participants

Retrospective analysis was conducted on the consecutive people who did total body measurement using DXA in the First Affiliated Hospital of Jinan University from July 2005 to October 2015. Their case files were reviewed. Those with diseases which might affect LBM were excluded. Overall 12,194 subjects were included. Information about the 10,683 subjects (2,987 males and 7,696 females) from July 2005 to November 2014 was used to establish the equations. The males were aged 18.0 to 97.9 and the average age was 53.9 years, while the females were aged 18.0 to 98.6 with an average age of 55.8 years. The subjects were regrouped into six subgroups according to their BMI: the male and female underweight subgroups (16kg/m^2^ ≤ BMI < 18.5kg/m^2^), the male and female normal weight subgroups (18.5kg/m^2^ ≤ BMI < 25 kg/m^2^), and the male and female overweight subgroups (25kg/m^2^ ≤ BMI < 40 kg/m^2^). Besides, the female subjects were reassigned into two subgroups: the premenopausal subgroup and the postmenopausal one. Information about the 1,511 subjects (395 males and 1,116 females) from December 2014 to October 2015 was used to verify the equations. The males were aged 18.4 to 91.9 and their average age was 57.8 years. The females were aged 18.0 to 94.4 with an average age of 58.7 years. These subjects were also regrouped and reassigned using the above method. Current standards of BMI formulated by WHO were used [18].

Inclusion criteria were as follows: (1) age ≥ 18y; (2) no significant weight change in the last three months; (3) being born and having been living in southern China.

Exclusion criteria were as follows: (1) BMI < 16 kg/m^2^ or BMI > 40 kg/m^2^ (people whose BMI is < 16 kg/m^2^ or > 40 kg/m^2^ have significantly different body composition and thus were excluded); (2) a history of weight loss surgery or regular physical exercise, such as bodybuilding; (3) a history of metabolic diseases that might affect body composition and bone metabolism, such as chronic obstructive pulmonary disease (COPD), thyroid disease, cancer, and diabetes; and (4) a history of using medications that might affect body composition and bone metabolism, like corticosteroids and testosterone.

### 2.2. Anthropometric Measurement

Height (cm) was measured to the nearest 0.1 cm without shoes using a wall-mounted stadiometer. Weight (kg) was measured to the nearest 0.1 kg with light clothing on. BMI was calculated with the equation: BMI (kg/m^2^) = weight/ (height/100)^2^.

### 2.3. LBM_DXA

Lunar Prodigy DXA bone densitometer (GE Healthcare, Madison, WI) was used. During total body measurement, the participants were asked to lie supine on the scanning bed with their arms at their sides straightly, palms down isolated from the body, feet neutral, and ankles strapped. The scanner was calibrated daily with quality control model provided by the manufacturer and the performance was monitored according to the quality assurance protocol. Scanning was not performed until all the assurance procedures were finished. LBM measured by DXA (LBM_DXA) was analyzed automatically by the built-in Prodigy enCORE software (v.10.50.086). The root-mean-square coefficient of variation (RMS-%CV), or the short-term precision of LBM, was 0.93% [19]. All operations were done by two trained and highly skilled operators and all scans were conducted according to the manufacturer's instructions. Analysis results showed that all the subjects' images met the requirements for measurement and analysis.

### 2.4. Statistical Analysis

Categorical and measurement data were analyzed using descriptive statistics. Measurement data were expressed as mean ± standard deviation. A *P* value of < 0.05 was considered to indicate statistical significance. Variance analysis was done to determine whether there were linear relationships between height, weight, BMI, and age and LBM_DXA. A histogram of standardized residuals was drawn with LBM_DXA being the dependent variable to find out whether the standardized residuals in each group were approximately normally distributed. LBM estimated by equations was recorded as LBM_PE.

Equations for each group were developed with LBM_DXA as the dependent variable and anthropometric measures (height, weight, BMI, and age) as the predictor variables. They were analyzed using stepwise multilinear regression with the inclusion criterion being *α* = 0.10 and the exclusion criterion being *α* = 0.11, and their coefficient of determination (R^2^) and standard error of estimation (SEE) values were recorded.

The equations were validated in two ways in each validation subgroup. Paired-sample* t*-test was done to analyze the differences between LBM estimated with the equations for all males or females and those estimated with the equation for each subgroup, while cross-validation was done by comparing LBM_PE with LBM_DXA. Cross-validation was carried out from three aspects. Firstly, paired-sample* t*-test was performed to analyze the differences between LBM_DXA and LBM_PE. Values of mean difference and *P* values were recorded. Secondly, linear regression was used to analyze the relationship between LBM_DXA and LBM_PE, and R^2^ and SEE were recorded. Thirdly, the agreement between LBM_PE and LBM_DXA was evaluated with Bland-Altman plots. Bias and 95% limits of agreement (LoA) between LBM_PE and LBM_DXA were calculated. Bias referred to the mean difference ($\overset{-}{d}$) [20]. A bias of zero indicated perfect agreement between LBM_PE and LBM_DXA. 95% LoA was set as 1.96 standard deviations (SD) above and below the mean difference ($\overset{-}{d}$ – 1.96SD to $\overset{-}{d}$ + 1.96SD).

Descriptive statistics, paired-sample* t*-test, and linear regression analysis were performed with SPSS 19.0. Bland-Altman analysis was done with MedCalc.

## 3. Results

### 3.1. Subjects' Characteristics

There were no significant differences in anthropometric measures (height, weight, BMI, and age) and LBM_DXA between the prediction participants and validation participants in both the male and female groups. All subjects' general information is presented in Table 1.

### 3.2. PEs for Males

#### 3.2.1. Development

In the male group, variance analysis results showed that F and *P* were 2,667.547 and 0.000, respectively. According to the criterion of *α* = 0.05, the relationships between LBM_DXA and age, height, weight, and BMI were liner ones. The histogram indicated nearly normal distribution. ANOVA revealed linear relationships between LBM_DXA and each anthropometric variable in the three subgroups, and the standardized residuals were approximately normally distributed.

Equation for all males (PE_M_) had the highest predictive ability (R^2^ = 0.782, SEE = 3.14kg). Weight, height, and BMI were positively correlated with LBM, while age was negatively correlated with it. BMI-subgrouping did not increase but slightly reduced the prediction accuracy of the equations (R^2^= 0.724 to 0.776, SEE = 2.77kg to 3.33kg).

*Equation for the Male Group *

(1) PEM:  LBM  kg=−25.498−0.051  age+0.312  height+0.263  weight+0.373  BMIR2=0.782,  SEE=3.14kg

*Equations for BMI-Based Subgroups*

(2) PEM-under:  LBM  kg=−5.382−0.037  age+0.154  height+0.502  weightR2=0.776,  SEE=2.77kgPEM-normal:  LBM  kg=−6.467−0.065  age+0.205  height+0.391  weightR2=0.724,  SEE=3.08kgPEM-over:  LBM  kg=−74.474−0.025  age+0.602  height+1.077  BMIR2=0.740,  SEE=3.33kg

#### 3.2.2. Validation

**Figure 1:**
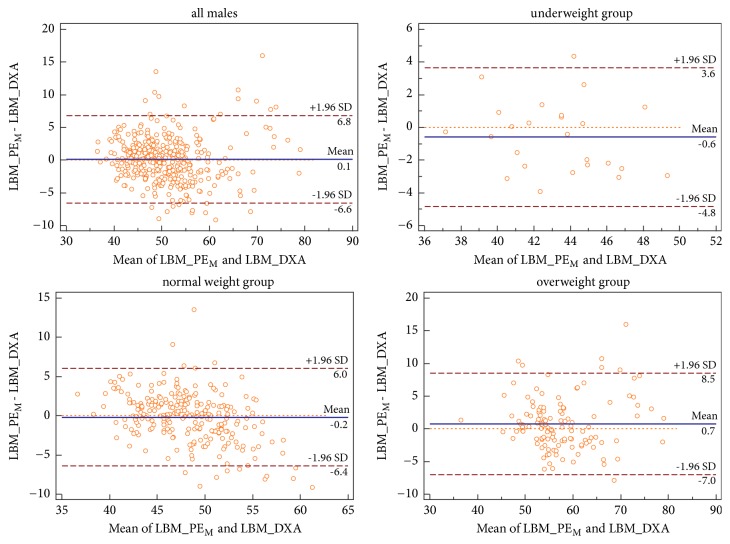
*Comparison between LBM_PE*_*M*_ *and LBM_DXA using Bland-Altman plots for males*. Abbreviations: PE_M_, prediction equation for all males; LBM, lean body mass; LBM_PE_M_, lean body mass calculated by PE_M_; LBM_DXA, lean body mass measured by dual-energy X-ray absorptiometry; SD, standard deviation.

There were statistically significant differences between LBM_PE_M_ and LBM_PE_M-under_, LBM_PE_M-normal,_ and LBM_PE_M-over_ while PE_M_ was used to predict LBM of each BMI-subgroup; however, the differences were very small (mean differences: 0.04kg to 0.13kg,* P* < 0.05; see Table 2). For this reason, cross-validation was only done on PE_M_. R^2^ and SEE of PE_M_ in the validation male subjects were similar to those in the prediction male subjects. Good agreement was observed between LBM_PE_M_ and LBM_DXA (bias = 0.05kg,* P* = 0.756, R^2^ = 0.803, and SEE = -3.35kg). In addition, the applicability of PE_M_ in the male subgroups was evaluated. There were no significant differences but good agreement between LBM_DXA and LBM_PE_M_ in each BMI-subgroup of males (bias: −0.59 to 0.73kg,* P* > 0.05, R^2^: 0.734 to 0.790, and SEE: 2.18kg to 3.77kg). Detailed information about difference and agreement between LBM_DXA and LBM_PE_M_ is listed in Table 2. Bland-Altman plots of PE_M_ are shown in Figure 1.

### 3.3. PEs for Females 

#### 3.3.1. Development

In the female group, variance analysis results showed that F and *P* were 5,930.758 and 0.000, respectively. Linear relationships were observed between LBM_DXA and age, height, weight, and BMI. The histogram was nearly in normal distribution. ANOVA showed that there were linear relationships between LBM_DXA and the anthropometric variables, and the standardized residuals were approximately normally distributed in all the subgroups.

Equation for all females (PE_F_) had higher predictability (R^2^ = 0.698, SEE = 2.43kg), though when compared with PE_M_ its R^2^ was slightly lower. Weight and height were positively correlated with LBM, which was similar to the situation in the male group, while BMI was negatively correlated with LBM and age was not introduced into PE_F_. Neither BMI-subgrouping nor menopause-subgrouping significantly improved the prediction accuracy of the equations (R^2^ = 0.662 to 0.733, SEE = 2.22kg to 2.68kg).

*Equation for the Female Group*

(3) PEF:  LBM  kg=8.032+0.534  weight+0.070  height−0.533  BMIR2=0.698,  SEE=2.43kg

*Equations for BMI-Based Subgroups*

(4) PEF-under:  LBM  kg=−17.742−0.010  age+0.235  height+0.300  weightR2=0.629,  SEE=2.22kgPEF-normal:  LBM  kg=18.856+0.634  weight−0.771  BMIR2=0.662,  SEE=2.38kgPEF-over:  LBM  kg=21.024+0.587  weight−0.019  age−0.697  BMIR2=0.687,  SEE=2.68kg

*Equation for Premenopausal Women*

(5) PEF-pre:  LBM  kg=−19.469+0.329  weight+0.234  height+0.031  ageR2=0.733,  SEE=2.44kg

*Equation for Postmenopausal Women*

(6) PEF-post:  LBM  kg=20.670+0.600  weight−0.712  BMI−0.021  ageR2=0.677,  SEE=2.40kg

#### 3.3.2. Validation

**Figure 2:**
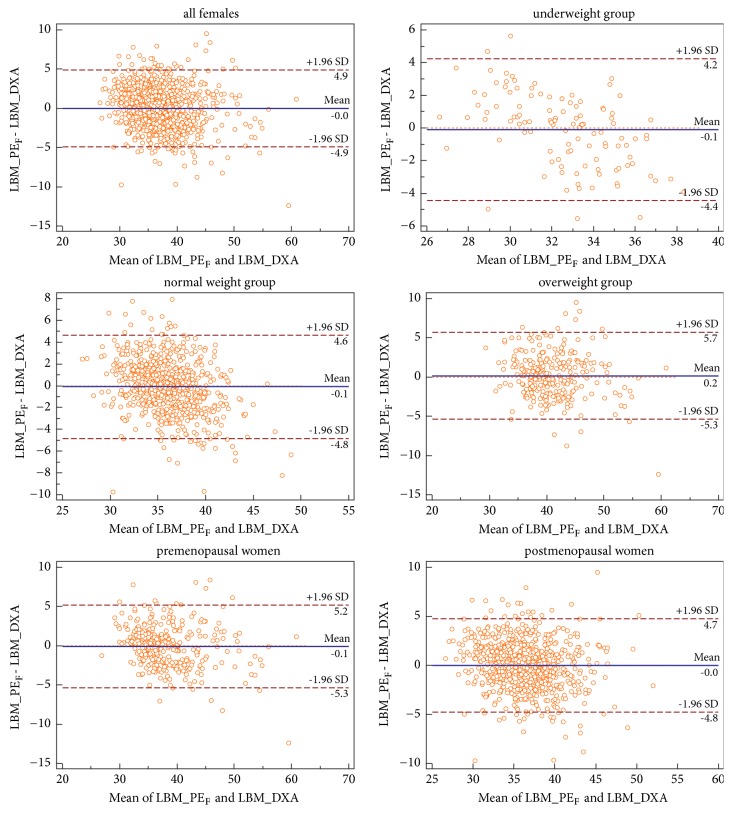
*Comparison between LBM_PE*_*M*_ *and LBM_DXA using Bland-Altman plots for females*. Abbreviations: PE_F_, prediction equation for all females; LBM, lean body mass; LBM_PE_F_, lean body mass calculated by PE_F_; LBM_DXA, lean body mass measured by dual-energy X-ray absorptiometry; SD, standard deviation.

There were statistically significant differences between LBM_PE_F_ and LBM_PE_F-under_, LBM_PE_F-normal_, LBM_PE_F-pre_, and LBM_PE_F-post_ while PE_F_ was used to predict LBM of each subgroup. However, the differences were very small (mean differences: −0.09kg to 0.63kg,* P* < 0.05; see Table 2). Therefore, cross-validation was only conducted on PE_F_. R^2^ and SEE of PE_F_ in the validation female subjects were similar to those in the prediction female subjects. Good agreement was observed between LBM_PE_F_ and LBM_DXA (bias = 0.03kg,* P* = 0.669, R^2^ = 0.734, and SEE = 2.49kg). In addition, the applicability of PE_F_ in the five subgroups was evaluated. There were no significant differences but good agreement between LBM_DXA and LBM_PE_F_ in the subgroups (bias = 0.10 to 0.17kg,* P* > 0.05, R^2^ = 0.668 to 0.791, and SEE = 2.21 kg to 2.79 kg). Details are listed in Table 2. Bland-Altman plots of PE_F_ are shown in Figure 2.

## 4. Discussion 

Sex-specific anthropometric equations of LBM are developed in this study with a large sample of healthy southern Chinese adults. LBM measured by DXA are considered as the standard ones. Validation results show that the equations boast high accuracy. We found that there was a correlation between BMI and LBM; however, the results demonstrate that there is no need for BMI-subgrouping and menopause-subgrouping while developing LBM prediction equations. These equations could be valuable tools to estimate LBM in large-scale epidemiologic studies and in remote areas where DXA or MRI are not available.

In addition to ethnicity, height, weight, and age are also important influencing factors of LBM. Studies have also found that LBM is also associated with BMI [21, 22]. In this study, height, weight, age, sex, and BMI are included in the PEs as predictor variables to analyze their effects on LBM. Yu et al. [7] found that introduction of biochemical variables into prediction equations could enhance the accuracy. In their study, the complex correlation coefficient, R^2^, increased from 90.7% to 91.9% after introduction of creatine kinase, lactate dehydrogenase, and high-sensitivity C-reactive protein as independent variables. However, insignificant changes of the values of R^2^ indicated that introduction of biochemical variables only had little effect on enhancing the prediction accuracy of equations, which made the effect not worth the cost and efforts.

Several anthropometric prediction equations of LBM have been developed, showing high predictability with high R^2^ ranging from 0.78 to 0.94 and low SEE ranging from 0.82kg to 3.61kg [5, 7, 14, 15]. However, no Chinese people were included as study subjects, which limited their use in China. Our equations enjoy high accuracy in estimating southern Chinese people's LBM (R^2^ = 0.782 and SEE = 3.14kg in PE_M_, R^2^ = 0.698 and SEE = 2.43kg in PE_F_). The relative proportion of variation explained by the prediction equation is greater for males than for females. Other studies also reported that equations for males' LBM had a higher prediction accuracy than those for females' [5, 23]. The gender difference probably reflects the differences in body composition between men and women. Males have much more LBM than females, while females have a greater range of variation in fat mass than males.

LBM prediction equations for BMI-based subgroups are designed to analyze the effect of BMI on LBM. However, BMI-subgrouping does not increase the accuracy of the equations but slightly decreases R^2^ in both male and female groups, which is believed to be the result of the narrow BMI ranges. This belief is confirmed by the fact that BMI cannot serve as a variable of both PE_M-under_ and PE_F-under_. Statistically significant differences are observed between LBM predicted by PE_M_/PE_F_ and LBM predicted by subgroup's equations in each BMI-subgroup, except the overweight female group. However, the differences are very small (mean difference: 0.04kg to 0.13kg in males and -0.09kg to 0.63kg in females,* P* < 0.05). In terms of biological variables, an error of more than 5% is of clinical significance [24]. An error of 0.13kg is negligible for a man with LBM of 50 kg, and an error of 0.63kg is also negligible for one woman with LBM of 35 kg. Therefore, there is no need for BMI-subgrouping in development of LBM prediction equations for southern Chinese people and no need to use different equation in people belonging to different BMI ranges. To confirm our conclusion, PE_M_ and PE_F_'s accuracy in the corresponding BMI-based subgroups is evaluated and results reveal good agreement between LBM_PE_M_/PE_F_ and LBM_DXA in each BMI-based subgroup with low bias, low SEE, and high R^2^.

Compared with PE_F_'s prediction accuracy (R^2^ = 0.698), PE _F-pre_'s is slightly higher (R^2^ = 0.733), while PE_F-post_'s is relatively lower (R^2^ = 0.677). This may be because estrogen levels decrease after menopause and there are differences in body composition between postmenopausal and premenopausal women. Some studies have suggested that decrease in the levels of estrogen and testosterone in postmenopausal women's serum may be a key cause of declining of LBM [25, 26]. Nevertheless, although PE _F-post_ has lower accuracy, further analysis shows that there is no need for menopause-subgrouping while designing LBM equation for southern Chinese women because of two facts. One is that the difference between PE_F_ and PE_F-pre_ and that between PE_F_ and PE_F-post_ are relatively small (about 0.1kg) and the other is that PE_F_ enjoys high accuracy in both premenopausal and postmenopausal women (bias = 0.08 kg, R^2^ = 0.791, and SEE = 2.68 kg in premenopausal women; bias = 0.01kg, R^2^ = 0.668, and SEE = 2.40kg in postmenopausal women).

Weight and height are positively correlated with LBM in both males (PE_M_) and females (PE_F_). However, BMI is positively correlated with LBM in males, while it is negatively correlated with LBM in females. Yu et al. [7] found that LBM increased with the decrease of BMI. Salamat et al. [14] found there was a positive correlation between LBM and BMI. Both the studies had very small samples, and neither of them reported the ratio of male subjects to female ones. The inconsistency among studies may result from differences in body composition between the two genders. BMI reflects heterogeneous regional body mass and composition scaling pattern [27].

Another interesting finding is that age is not an influencing factor of females' LBM, while it is negatively correlated with males' LBM. Some studies reported that both males and females experienced age-related decease in LBM and that age had more impacts on males' LBM than on females' [5, 15, 23]. Heymsfield et al. [22] found that age was an important negative predictor of skeletal muscle mass after control of height in men but not in women. Our previous study also found that the patterns of age-related LBM changes were different between Chinese men and women. It was reported that Chinese males' lean mass index was negatively correlated with age, while no correlation was observed between age and lean mass index in Chinese females [28]. These sex-related differences in body composition may mainly result from sex steroid hormones, which promote sexual dimorphism during pubertal development [29].

Our equations are developed using simplest anthropometric measurements, which can be made quickly in epidemiologic settings. The large sample and a broad range of age and BMI guarantee their high accuracy. It should be noted that, although they have been validated and have high accuracy in epidemiological settings, they are not accurate enough for clinical or individual use. The 95% LoA is (-6.6kg, 6.8kg) in males and (-4.9kg, 4.9kg) in females in this study, which is similar to the study by Lee et al. [5]. Bland-Altman plots show that the difference between LBM_DXA and LBM_PE can be as high as 15.96kg in males, and LBM_DXA of that individual is 63.20kg, which means that the equation overestimates his LBM by about 25%.

This study has several limitations. The data about body composition were collected only from the First Affiliated Hospital of Jinan University. The study should have included people from other research centers in order to make the conclusions suitable for each southern Chinese adult. Secondly, differences in scanning pattern, software version, and calibration method among different DXA by different manufacturers might result in measurement errors [30]. Lunar Prodigy DXA was used in this study. Thirdly, most of the participants in this study are patients in hospital. However, patients with diseases which might affect bone density and body composition were excluded. Besides, it was reported that the use of other anthropometry measurements such as hip or waist circumference can improve the performance of LBM prediction equations [5, 15]; however, they were not included in this study. Future studies are needed to eliminate these limitations.

## 5. Conclusions 

Gender-specific prediction equations for southern Chinese people's LBM are developed and verified with a large sample in this study. They can be used in epidemiological settings to evaluate body composition. BMI was related to LBM content; however, there is no need for further group based on BMI or menopause while developing LBM questions.

## Figures and Tables

**Table 1:** Participants' characteristics (n=12,194).

	Prediction participants		Validation participants	
	Males	Females	Males	Females
n	2,987	7,696	395	1,116
LBM_DXA (kg)	50.0±6.7*∗*	36.5±4.4^#^	50.8±7.5	37.0±4.8
Age (year)	53.9±17.7*∗*	55.8±15.5^#^	57.8±18.0	58.7±16.2
Height (cm)	167.9±6.3*∗*	156.7±5.4^#^	168.2±6.9	156.3±6.0
Weight (kg)	65.3±12.5*∗*	55.2±9.4^#^	68.1±15.4	56. 7±11.2
BMI (kg/m^2^)	23.1±3.7*∗*	22.4±3.4^#^	23.9±4.3	23.2±4.0
16–18.49	328 (11.0%)	885 (11.5%)	25 (6.3%)	112 (10.0%)
18.5–24.99	1,823 (61.0%)	5,238 (68.1%)	245 (62.0%)	718 (64.3%)
25–39.99	836 (28.0%)	1,573 (20.4%)	125 (31.6%)	286 (25.6%)

Grouping variables were expressed as frequency (rate), while numerical values were expressed as mean ± standard deviation.

*∗ P* > 0.05, compared with males of validation participants. ^#^*P* > 0.05, compared with females of validation participants.

Abbreviations: LBM_DXA, lean body mass measured by dual-energy X-ray absorptiometry.

**Table 2:** Comparison between LBM_PE and LBM_DXA in the validation subjects (n = 1,511).

	Groups	n	LBM_PE_M/F_ (kg)	LBM by equations for subgroups (kg)	LBM_DXA (kg)	Bias (kg)^a^	*P*^b^	95% LoA^c^	R^2^	SEE
Males	All	395	50.87±7.46	/	50.82±7.53	0.05±3.42	0.756	-6.6, 6.8	0.803	3.35
	Underweight	25	42.99±2.87*∗*	42.88±2.72	43.59±3.33	-0.59±2.16	0.183	-4.8, 3.6	0.790	2.18
	Normal weight	245	48.06±3.97*∗*	48.02±4.02	48.24±5.22	-0.17±3.16	0.387	-6.4, 6.0	0.734	3.16
	Overweight	125	58.06±7.91*∗*	57.93±7.48	57.33±7.61	0.73±3.97	0.062	-7.0, 8.5	0.757	3.77

Females	All	1,116	36.92±4.38	/	36.95±4.83	-0.03±2.50	0.669	-4.9, 4.9	0.734	2.49
	Underweight	112	32.51±2.17*∗*	31.28±2.45	32.61±3.21	-0.10±2.21	0.633	-4.4, 4.2	0.727	2.21
	Normal weight	718	35.97±2.94*∗*	36.00±2.94	36.08±3.67	-0.10±2.42	0.259	-4.8, 4.6	0.748	2.42
	Overweight	286	41.02±4.95^#^	41.03±4.94	40.85±5.36	0.17±2.81	0.308	-5.3, 5.7	0.731	2.79
	Premenopausal	339	38.30±5.26*∗*	38.39±5.25	38.38±5.85	-0.08±2.67	0.559	-5.3,5.2	0.791	2.68
	Postmenopausal	777	36.32±3.78*∗*	36.22±3.70	36.33±4.16	-0.01±2.43	0.917	-4.8, 4.7	0.668	2.40

LBM, lean body mass; PE_M_, equation for all males; PE_F_, equation for all females; SEE, standard error of estimation; R^2^, coefficient of determination; LBM_PE, lean body mass calculated by prediction equations; LBM_DXA, lean body mass measured by dual energy X-ray absorptiometry.

^a^ Bias shows differences between LBM_PE and LBM_DXA and results are expressed as mean ± standard deviation.

^b^ Paired sample t-test is performed to evaluate the differences between LBM_PE and LBM_DXA. *P* < 0.05 indicates that the difference is statistically significant.

^c^ 95% LoA: 95% limits of agreement for mean difference; limits of agreement = $\overset{-}{d}$ - 1.96 standard deviations – $\overset{-}{d}$ + 1.96 standard deviations.

*∗ P* < 0.05, compared with LBM by equations for the subgroups; ^#^*P* > 0.05, compared with LBM by equations for the subgroups.

## Data Availability

The data used to support the findings of this study are available from the corresponding author upon request.

## References

[1] Ward L. C. (2018). Human body composition: yesterday, today, and tomorrow. *European Journal of Clinical Nutrition*.

[2] Pin F., Couch M. E., Bonetto A. (2018). Preservation of muscle mass as a strategy to reduce the toxic effects of cancer chemotherapy on body composition. *Current Opinion in Supportive and Palliative Care*.

[3] Hopkins J. J., Sawyer M. B. (2017). A review of body composition and pharmacokinetics in oncology. *Expert Review of Clinical Pharmacology*.

[4] Wahl R. L., Jacene H., Kasamon Y. (2009). From RECIST to PERCIST: evolving considerations for PET response criteria in solid tumors. *Journal of Nuclear Medicine : Official Publication, Society of Nuclear Medicine*.

[5] Lee D. H., Keum N., Hu F. B. (2017). Development and validation of anthropometric prediction equations for lean body mass, fat mass and percent fat in adults using the National Health and Nutrition Examination Survey (NHANES) 1999-2006. *British Journal of Nutrition*.

[6] Lemos T., Gallagher D. (2017). Current body composition measurement techniques. *Current Opinion in Endocrinology, Diabetes and Obesity*.

[7] Yu S., Visvanathan T., Field J. (2013). Lean body mass: the development and validation of prediction equations in healthy adults. *BMC pharmacology & toxicology*.

[8] Wang J., Streja E., Rhee C. M. (2016). Lean body mass and survival in hemodialysis patients and the roles of race and ethnicity. *Journal of Renal Nutrition*.

[9] Shah A. D., Kandula N. R., Lin F. (2016). Less favorable body composition and adipokines in South Asians compared with other US ethnic groups: Results from the MASALA and MESA studies. *International Journal of Obesity*.

[10] Gallagher D., Heymsfield S. B., Heo M., Jebb S. A., Murgatroyd P. R., Sakamoto Y. (2000). Healthy percentage body fat ranges: an approach for developing guidelines based on body mass index. *American Journal of Clinical Nutrition*.

[11] Deurenberg-Yap M., Schmidt G., van Staveren W. A., Deurenberg P. (2000). The paradox of low body mass index and high body fat percentage among Chinese, Malays and Indians in Singapore. *International Journal of Obesity and Related Metabolic Disorders : Journal of the International Association for the Study of Obesity*.

[12] Deurenberg P., Weststrate J. A., Seidell J. C. (1991). Body mass index as a measure of body fatness: age- and sex-specific prediction formulas. *British Journal of Nutrition*.

[13] Heitmann B. L. (1990). Evaluation of body fat estimated from body mass index, skinfolds and impedance. a comparative study. *European Journal of Clinical Nutrition*.

[14] Salamat M. R., Shanei A., Salamat A. H. (2015). Anthropometric predictive equations for estimating body composition. *Advanced Biomedical Research*.

[15] Kulkarni B., Kuper H., Taylor A. (2013). Development and validation of anthropometric prediction equations for estimation of lean body mass and appendicular lean soft tissue in Indian men and women. *Journal of Applied Physiology*.

[16] Wen X., Wang M., Jiang C.-M., Zhang Y.-M. (2011). Anthropometric equation for estimation of appendicular skeletal muscle mass in Chinese adults. *Asia Pacific Journal of Clinical Nutrition*.

[17] Morgan D. J., Bray K. M. (1994). Lean body mass as a predictor of drug dosage: implications for drug therapy. *Clinical Pharmacokinetics*.

[18] WHO Expert Consultation (2004). Appropriate body-mass index for Asian populations and its implications for policy and intervention strategies. *The Lancet*.

[19] Guo B., Xu Y., Gong J., Tang Y., Shang J., Xu H. (2015). Reference data and percentile curves of body composition measured with dual energy X-ray absorptiometry in healthy Chinese children and adolescents. *Journal of Bone and Mineral Metabolism*.

[20] Bland J. M., Altman D. G. (2007). Agreement between methods of measurement with multiple observations per individual. *Journal of Biopharmaceutical Statistics*.

[21] Murphy R. A., Ip E. H., Zhang Q. (2014). Transition to sarcopenia and determinants of transitions in older adults: a population-based study. *The Journals of Gerontology. Series A, Biological Sciences and Medical Sciences*.

[22] Heymsfield S. B., Gallagher D., Mayer L., Beetsch J., Pietrobelli A. (2007). Scaling of human body composition to stature: New insights into body mass index. *American Journal of Clinical Nutrition*.

[23] Al-Gindan Y. Y., Hankey C., Govan L., Gallagher D., Heymsfield S. B., Lean M. E. J. (2014). Derivation and validation of simple equations to predict total muscle mass from simple anthropometric and demographic data. *American Journal of Clinical Nutrition*.

[24] Khoo B. C. C., Beck T. J., Qiao Q.-H. (2005). In vivo short-term precision of hip structure analysis variables in comparison with bone mineral density using paired dual-energy X-ray absorptiometry scans from multi-center clinical trials. *Bone*.

[25] Messier V., Rabasa-Lhoret R., Barbat-Artigas S., Elisha B., Karelis A. D., Aubertin-Leheudre M. (2011). Menopause and sarcopenia: a potential role for sex hormones. *Maturitas*.

[26] Geel T. A. V., Geusen P. P., Winkens B., Sels J.-P. J. E., Dinant G.-J. (2009). Measures of bioavailable serum testosterone and estradiol and their relationships with muscle mass, muscle strength and bone mineral density in postmenopausal women: a cross-sectional study. *European Journal of Endocrinology / European Federation of Endocrine Societies*.

[27] Schuna Jr. J. M., Peterson C. M., Thomas D. M. (2015). Scaling of adult regional body mass and body composition as a whole to height: Relevance to body shape and body mass index. *American Journal of Human Biology*.

[28] Xiao Z., Guo B., Gong J. (2017). Sex- and age-specific percentiles of body composition indices for Chinese adults using dual-energy X-ray absorptiometry. *European Journal of Nutrition*.

[29] Wells J. C. (2007). Sexual dimorphism of body composition. *Best Practice & Research. Clinical Endocrinology & Metabolism*.

[30] Bazzocchi A., Ponti F., Albisinni U., Battista G., Guglielmi G. (2016). DXA: technical aspects and application. *European Journal of Radiology*.

